# A density gradient centrifugation method for rapid separation of nanoTiO_2_ and TiO_2_ aggregates from microalgal cells in complex mixtures with mercury

**DOI:** 10.1016/j.mex.2020.101057

**Published:** 2020-09-07

**Authors:** Mengting Li, Vera I. Slaveykova

**Affiliations:** Environmental Biogeochemistry and Ecotoxicology, Department F.-A. Forel for environmental and aquatic sciences, Earth and Environmental Sciences, Faculty of Sciences and Institute for Environmental Science, University of Geneva, Uni Carl Vogt, Bvd Carl-Vogt 66, CH-1211 Geneva 4, Switzerland

**Keywords:** Green microalgae, Nanoparticles, Density gradient centrifugation, Mixtures

## Abstract

In natural environment, the microorganisms are exposed to complex mixtures of contaminants, including manufactured nanoparticles and their aggregates. Evaluation of the toxicant accumulation in biota exposed to such cocktails is a challenging task because the microorganisms need to be separated from nanomaterial aggregates often of a comparable size. We propose a method for separation of TiO_2_ aggregates from green microalga *Chlamydomonas reinhardtii* and subsequent determination of cellular Hg concentration in algae exposed to mixture of Hg with nanoTiO_2_, known also to adsorb Hg. The method is based on differences in specific weight of algae and TiO_2_ aggregates, using medium speed centrifugation on a step gradient of sucrose. The efficiency of the separation method was tested with nanoTiO_2_ of three different primary sizes at four concentrations: 2, 20, 100 and 200 mg L^−1^. The method gives a possibility to separate nanoTiO_2_ and their aggregates from the algae with a mean recovery of 83.3% of algal cells, thus allowing a reliable determination of Hg accumulation by microalgae when co-exposed to Hg and nanoTiO_2_.

• A rapid and reliable method to separate algal cells and nanoparticle aggregates of comparable size.

• A method to measure the cellular amount of Hg in green alga co-exposed to Hg and nanoTiO_2_.

## Graphical abstract

**Figure:**
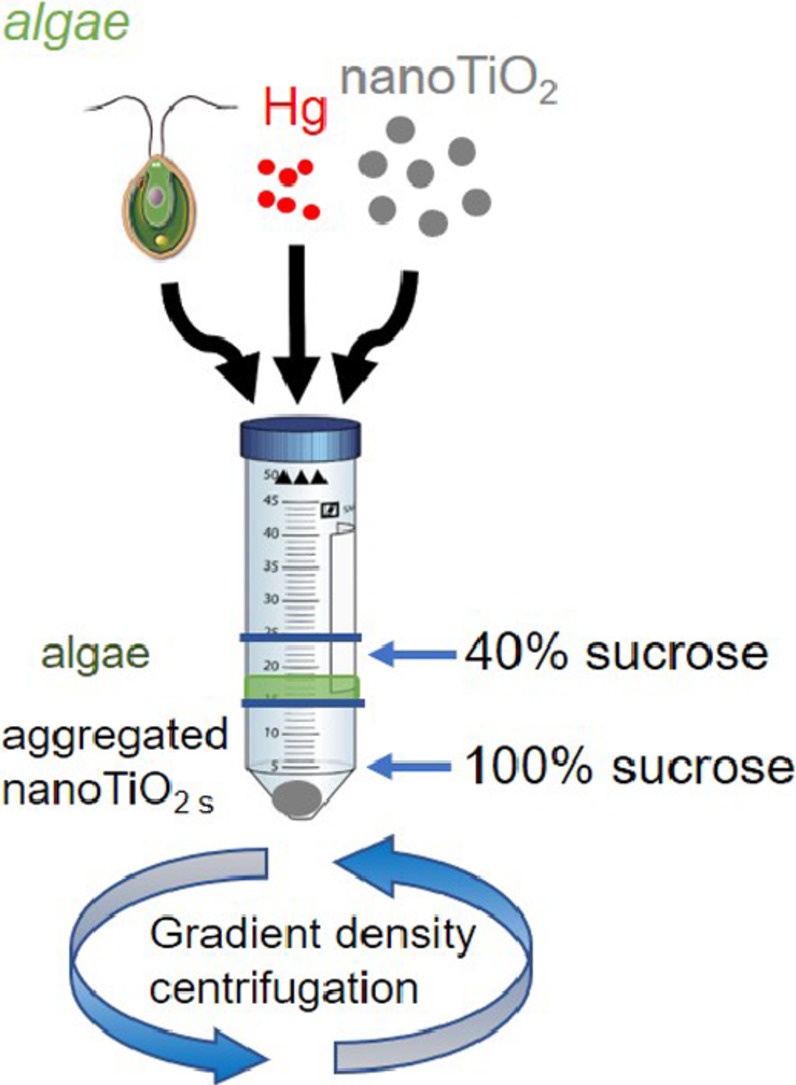

Specifications table

**Table:** 

Subject Area	Environmental Science
More specific subject area	*Environmental (nano)toxicology*
Method name	*A density gradient centrifugation method for rapid separation of nanoTiO_2_ and TiO_2_ aggregates from microalgal cells in complex mixtures with mercury*
Name and reference of original method	*–*
Resource availability	*–*

## 1 Method details

### 1.1 Background

In the aquatic environments, contaminants are found as complex mixtures [7,10]. Due to the extensive use of engineered nanomaterials (ENMs) in industry and consumer products, a variety of ENMs is inevitably disposed or released into the environment [5,6], including titanium dioxide nanoparticles (nanoTiO_2_) [11,12]. Thus, it is pressing to determine the interactions and effects of nanoTiO_2_ in mixtures because of their high reactivity. NanoTiO_2_ is known to adsorb the dissolved contaminants [3,^4^,13]. Our previous work found that inorganic Hg (IHg) could significantly adsorbed to the nanoTiO_2_ materials within 2 h [8]. Determining the bioaccumulation potential of the contaminants coexisting with ENMs is an important component of the hazard assessment of chemical mixtures. This requires quantification of cellular contaminants, after effective removal of the unbound or loosely bound nanomaterials from the organisms which may cause overestimation of the content of cellular contaminants. However, in the conventional differential centrifugation, where particles are separated based on their size and density, the aggregates of nanoTiO_2_ are expected to sediment along with algal cells. Filtration is not an option due to the presence of TiO_2_ aggregates of size similar to those of the algal cells in suspension. Here we present a novel method for separation of the nanoTiO_2_and their aggregates from green microalga *Chlamydomonas reinhardtii* and subsequent determination of the bioaccumulated Hg during exposure to mixtures of Hg and nanoTiO_2_. This method is adaptation of a previous methodology successfully used to separate bacterium *Pseudomonas aeruginosa* from unbound multiwall carbon nanotubes (MWCNTs) and MWCNT aggregates [9].

### 1.2 Materials and equipment

• Wild-type *C. reinhardtii* (CPCC11, Canadian Phycological Culture Centre, Waterloo, Canada)
• Powdered nanoscale TiO_2_ particles with different structure and size (anatase, 5 nm (A5), anatase, 15 nm (A15) and anatase/rutile, 20 nm (AR20), Nanostructured & Amorphous Materials, Inc., USA)
• Sucrose (analytical grade)
• HgCl_2_ standard solution (1.0 g L^−1^, Sigma-Aldrich, Buchs, Switzerland)
• Algal exposure medium (8.2 × 10^−4^ M CaCl_2_•2H_2_O, 3.6 × 10^−4^ M MgSO_4_•7H_2_O, 2.8 × 10^−4^ M NaHCO_3_, 1.0 × 10^−4^ M KH_2_PO_4_ and 5.0 × 10^−6^ M NH_4_NO_3_, pH 7.0 ± 0.1)
• Acid-washed and autoclaved 15 and 50 mL centrifuge tubes
• Cooling centrifuge for cell harvesting
• Flow cytometer (FCM, BD Accuri 6, BD Biosciences, San Jose, CA)
• Advanced Hg Analyzer AMA 254 (Altec s.r.l., Czech Republic)

### 1.3 Procedure

The experimental procedure consists of three steps: (i) Exposure of algal cells to mixtures containing mercury and nanoTiO_2_; (ii) Separation of algal cells from TiO_2_ aggregates based on two‐step centrifugation and (iii) Determination of the cellular Hg in alga. Here we present in details the steps (ii) and (iii).

**Fig. 1:**
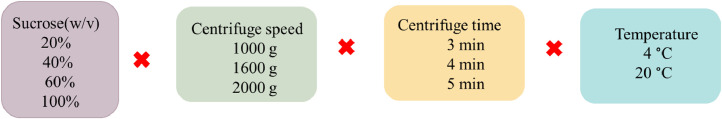
Experimental conditions for density gradient centrifugation tested to select the best combinations of sucrose density, centrifuge speed, time, and temperature for nanoTiO_2_ separation from algae.

**Fig. 2:**
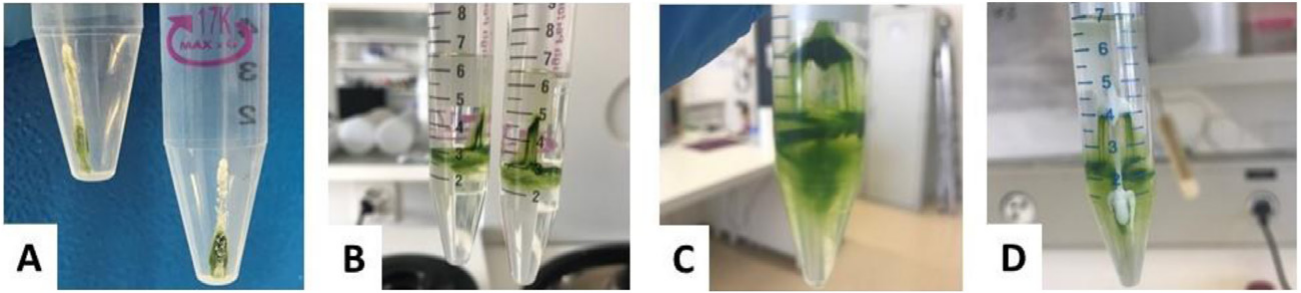
Examples of unsuccessful tests. (A) TiO_2_ aggregates sediment along with algal cells during conventional differential centrifugation step; (B) centrifugation time is too short to get all the alga settle down during density gradient centrifugation step; (C) density of sucrose was too low; (D) density of sucrose was too high, which prevented the TiO_2_ aggregates to sediment.

Separation of algal cells from TiO_2_ aggregates is based on two‐step centrifugation: (i) differential centrifugation; and (ii) density gradient centrifugation in sucrose solutions. The variables to optimize include sucrose density gradient, centrifuge speed and time, and temperature. The selection of optimal conditions depends on the size and density of the materials being analyzed. According to the published literature, marine plankton is localize in an approximately 25–60% sucrose layer, meaning that their buoyant density is equal to the density of sucrose in this concentration range [2]. Theoretically, alga will settle down faster than individual nanoparticles but slower than nanoparticle aggregates. Thus, the localization of *C. reinhardtii* in sucrose was verified in 20–60% sucrose in nanoTiO_2_-free exposure medium. The localization of nanoTiO_2_ in sucrose was tested experimentally in 20–100% sucrose (Fig. 1), employing nanoTiO_2_ suspensions in alga-free exposure medium. These two series of tests allowed to determine the optimal sucrose gradient concentrations. Because the ultimate goal was to assess bioaccumulation of Hg to *C. reinhardtii,* the duration of the separation procedure should be kept as short as possible (3–5 min). Two centrifugation temperatures were tested: 4 and 20 °C. To confirm that the cellular content of Hg was not affected by the separation procedure, the amounts of Hg accumulated in *C. reinhardtii* in the absence of nanoTiO_2_ were quantified by atomic absorption spectrometry using the Advanced Hg Analyzer AMA 254 (Altec s.r.l., Czech Republic) before and after the separation by differential centrifugation followed by density gradient centrifugation. Some examples of different tentative for separation (Fig. 2) clearly demonstrated the need of the performing differential centrifugation (Fig. 2A) and careful optimization of the experimental condition of density gradient centrifugation.

**Fig. 3:**
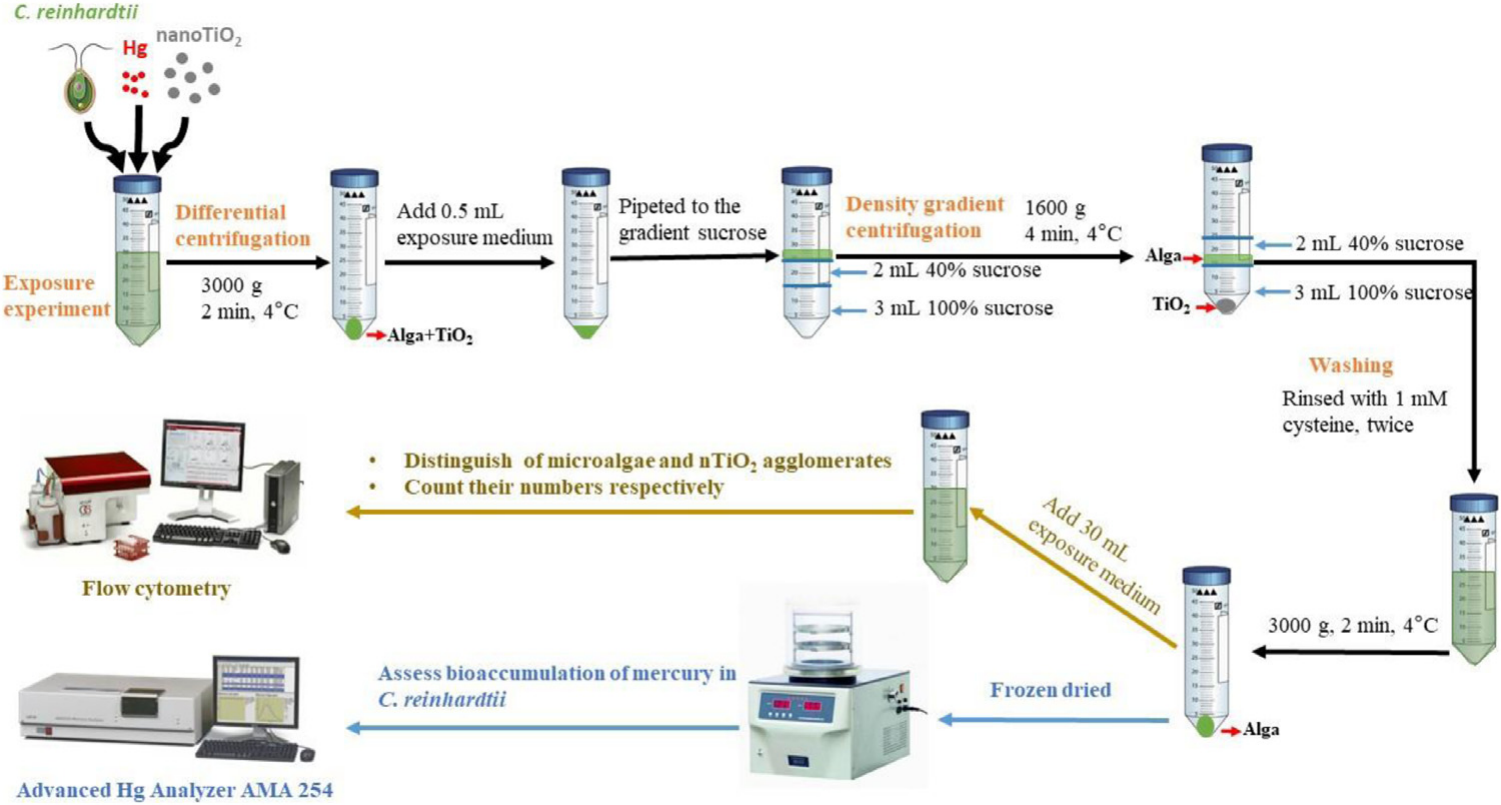
Summary of developed procedure for separation of algal cells from nanoTiO_2_ and TiO_2_ aggregates using density gradient centrifugation.

Based on a number of different tests with 10% step increase of the sucrose percentage, 40% (w/v) sucrose was chosen for separation of individually dispersed nanoTiO_2_ and algal cells. Under this condition almost all algae were localized in sucrose layer. 100% (w/v) sucrose was chosen for separation of algal cells and compact TiO_2_ aggregates, as TiO_2_ aggregates concentrated exclusively in this sucrose layer. After centrifugation for 4 min at 1600 *g* at 4 °C, the upper layer of 100% sucrose containing algae could be clearly distinguished by its green color. During the tests, algal cell numbers and nanoTiO_2_ particle numbers were determined by flow cytometry. The method summarized in Fig. 3 and described herein in detail is used to assess the bioaccumulation of Hg by *C. reinhardtii* exposed to the mixtures of Hg and nanoTiO_2_.

#### 1.3.1 Preparation

1. Stock solution of 2.0 g L^−1^ of nanoTiO_2_ were prepared by dispersing nanoparticles in ultrapure water and applying sonication for 10 min (50 W L^−1^ at 40 kHz), and a further 10 min sonication was conducted immediately before dosing.
2. All the glassware was soaked for at least 24 h in 5% v/v HNO_3_, rinsed three times with ultrapure water and autoclaved prior use.

#### 1.3.2 Exposure of *C. reinhardtii* to Hg, nanoTiO_2_, and their mixtures

3. For each trial, the algal cells at mid-exponential growth phase were re-suspended (~10^6^ cells mL^−1^) in 30 mL exposure medium which contained Hg (10^−9^ M) or mixtures of nanoTiO_2_ (2, 20, 100, and 200 mg L^−1^) and Hg (10^−9^ M) for 24 h. Cells exposed in the absence of Hg and nanoTiO_2_ were used as control. Exposures and analyses were performed on three biological replicates.

#### 1.3.3 Differential centrifugation

4. 30 mL suspensions were centrifuged at 3000 g for 2 min at 4 °C to collect the pellet containing algae and nanoTiO_2_, to get enough algal cells for quantitative analysis of their intracellular mercury.
5. Wash the cells twice with 30 mL 10^−3^ M ethylene diamine tetraacetic acid (EDTA; Sigma-Aldrich, Buchs, Switzerland) and 10^−3^ M cysteine (Sigma-Aldrich, Buchs, Switzerland) [1], respectively, by 1 min vortexing followed by centrifugation at 3000 *g* for 2 min at 4 °C to remove the extracellular loosely bound nanoTiO_2_ and Hg.
6. Pellet was resuspended in 0.5 mL of exposure medium, vortexed for 1 min to obtain a homogeneous suspension.

#### 1.3.4 Density gradient centrifugation

7. 2 mL of 40% sucrose were pipetted carefully over 3 mL of 100% sucrose into sterile conical polypropylene centrifuge tubes of 15 mL.
8. Algal suspensions obtained in step 5 were carefully pipetted over the sucrose gradient of 40% and 100% (w/v) and transferred to sterile conical polypropylene centrifuge tubes of 15 mL.
9. Tubes were centrifuged for 4 min at 1600 *g* at 4 °C.
10. Pellet was resuspended in 0.5 mL of exposure medium, vortexed for 1 min to obtain a homogeneous suspension.

### 1.4 Separation method validation

**Fig. 4:**
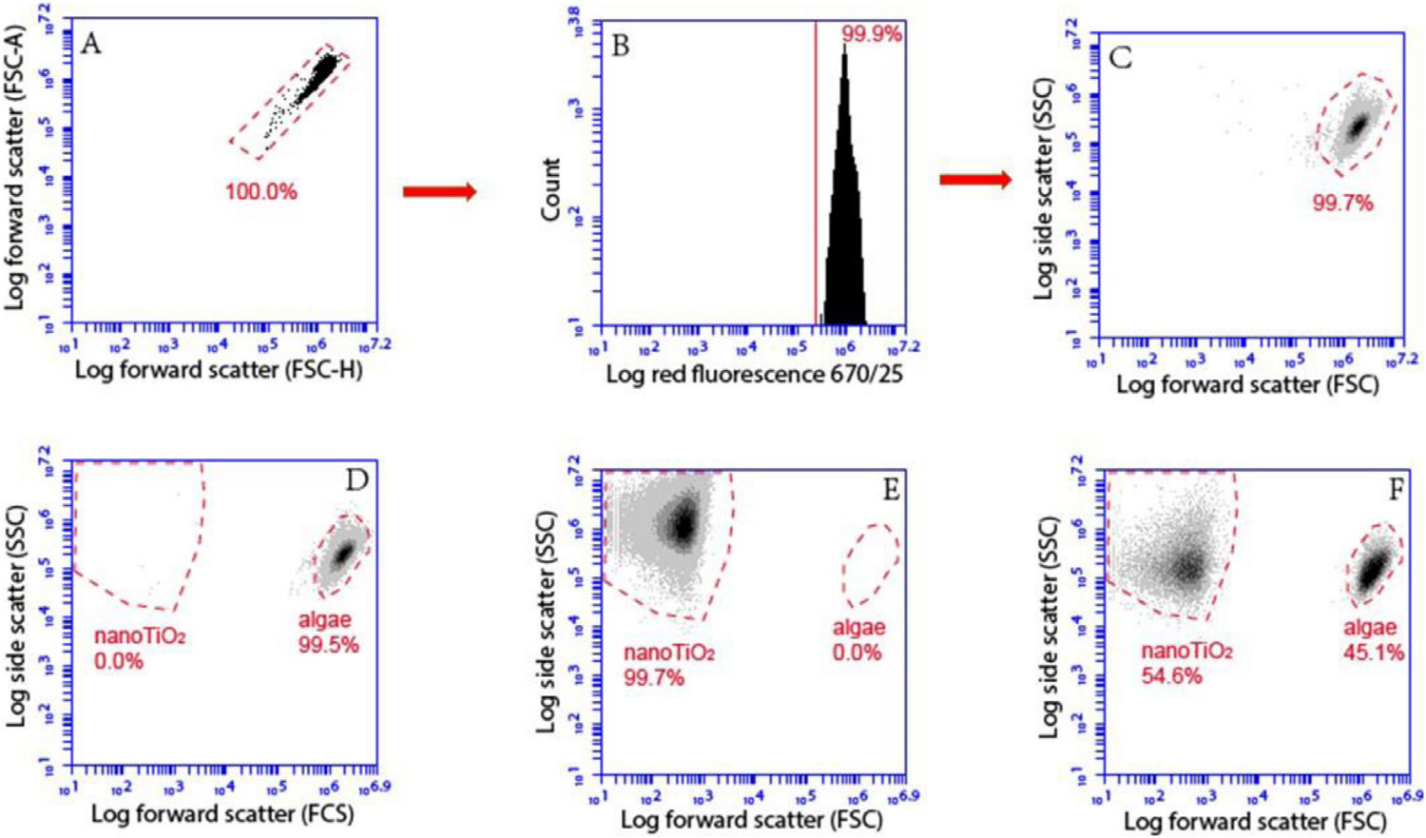
Schematic representation of the FCM data analysis procedure used to define the gate corresponding to algae and nanoTiO_2_ aggregates. As a first step FSC-H/FSC-A dot-plot (A) was used to remove eventual instrument background. Then signals present in the gated region were plotted in a count versus red fluorescence plot (B) and SSC/FSC dot-plot (C) to verify cellular characteristics of size, granularity and chlorophyll autofluorescence. (D) cytogram of algal suspension (1.0 × 10^6^ cells/mL) in the absence of nanoTiO_2_, (E) cytogram of 200 mg L^−1^ nanoTiO_2_ suspension only (F) cytogram of mixture containing 1.0 × 10^6^ cells/mL algaeand 200 mg L^−1^ nanoTiO_2_.

To evaluate the efficiency of the separation of the microalgae from the aggregates of nanoTiO_2_, the cell numbers and the numbers of TiO_2_ aggregates were determined by using flow cytometry before and after density gradient centrifugation. 488 nm argon excitation laser and fluorescence detection channel with band pass emission filters at a long pass emission filter for > 670 nm (FL3) were used. Data acquisition and analysis were performed with the BD Accuri C6 Software 264.15. The primary threshold was set to 20,000 events on FSC-H. Algal cells were discriminated from nanoTiO_2_ aggregates applying the gating strategy shown on Fig. 4. The log FSC-H versus log FSC-A dot-plot was used first to remove cell doublets or artefacts. Then two different plots (log SSC-A versus log FSC-A and count versus log FL3) were used to distinguish algal cells and TiO_2_ aggregates based on the difference in their size and chlorophyll autofluorescence specific for alga.

**Fig. 5:**
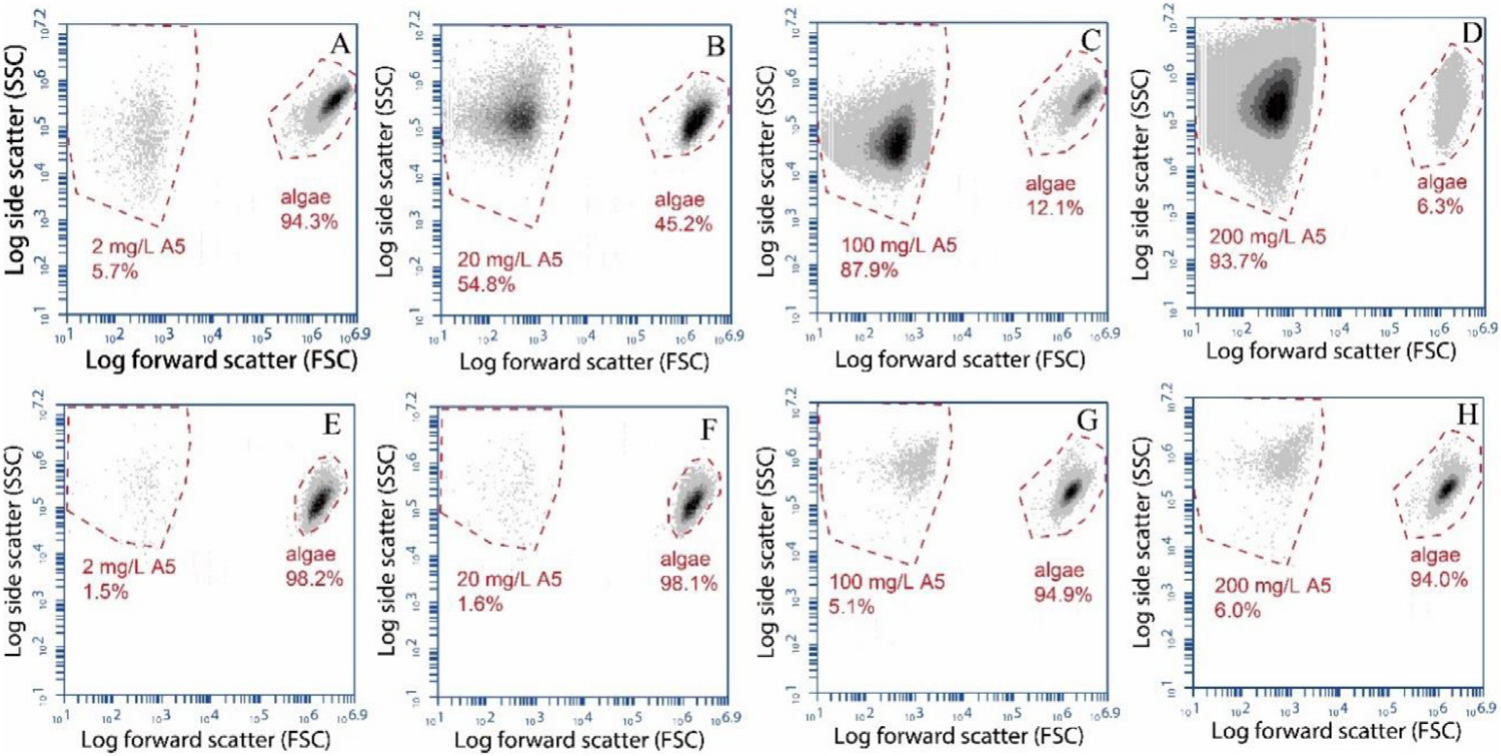
FCM results of *C. reinhardtii* mix with 2 mg L^−1^ (A), 20 mg L^−1^ (B), 100 mg L^−1^ (C), and 200 mg L^−1^ (D) A5, taken before (A, B, C, D) and after (E, F, G, H) density gradient centrifugations.

After density gradient centrifugation, the fractions collected from the region containing the green colored bands were resuspended in the same volume of initial algal suspensions and the cell densities were measured by FCM. The numbers of algae and TiO_2_ particles were counted before and after density gradient centrifugation step (Fig. 5). Before the density gradient centrifugation, the ratios between alga and 2–200 mg L^−1^ TiO_2_ were 16.54, 0.82, 0.14, and 0.07, respectively (Fig. 4 A–D). Our data show that above 98% of the three types of nanoTiO_2_ with a large range of concentrations (2-200 mg L^−1^) were removed from the algal pellet (Fig. 4E–H). A mean recovery of 83.3% algal cells was found by flow cytometry measuring cell density in suspensions after density gradient centrifugation and prior centrifugation. Initial density was (1.0 ± 0.056) x10^6^ cells mL^−1^.

#### 1.4.1 Quantification of Hg accumulation by *C. reinhardtii* in complex mixtures with nanoTiO_2_

After 24 h exposure, 30 mL of algal suspensions were centrifuged using the density gradient centrifugation method developed. Algal pellets were stored frozen at −20 °C until analysis in clean centrifugation flasks. The samples were dried in a freeze-dryer for at least 24 h. Freeze-dried samples were weighed (±0.00001 *g*) and the amount of accumulated Hg were determined by atomic absorption spectrometry using the Advanced Hg Analyzer AMA 254 (Altec s.r.l., Czech Republic). The accuracy of the measurements was checked by analyzing the certified reference material (CRM) MESS-3 (100 ± 0.1% recovery). Initial THg concentrations in the exposure medium were determined using MERX® Automated Total Mercury Analytical System (Brooks Rand Instruments, Seattle, WA, USA). Detection limit was 0.03 ng THg L^−1^. The accuracy of THg measurements was verified by analyzing the CRM ORMS-5 (116.0 ± 3.5% recovery).

**Fig. 6:**
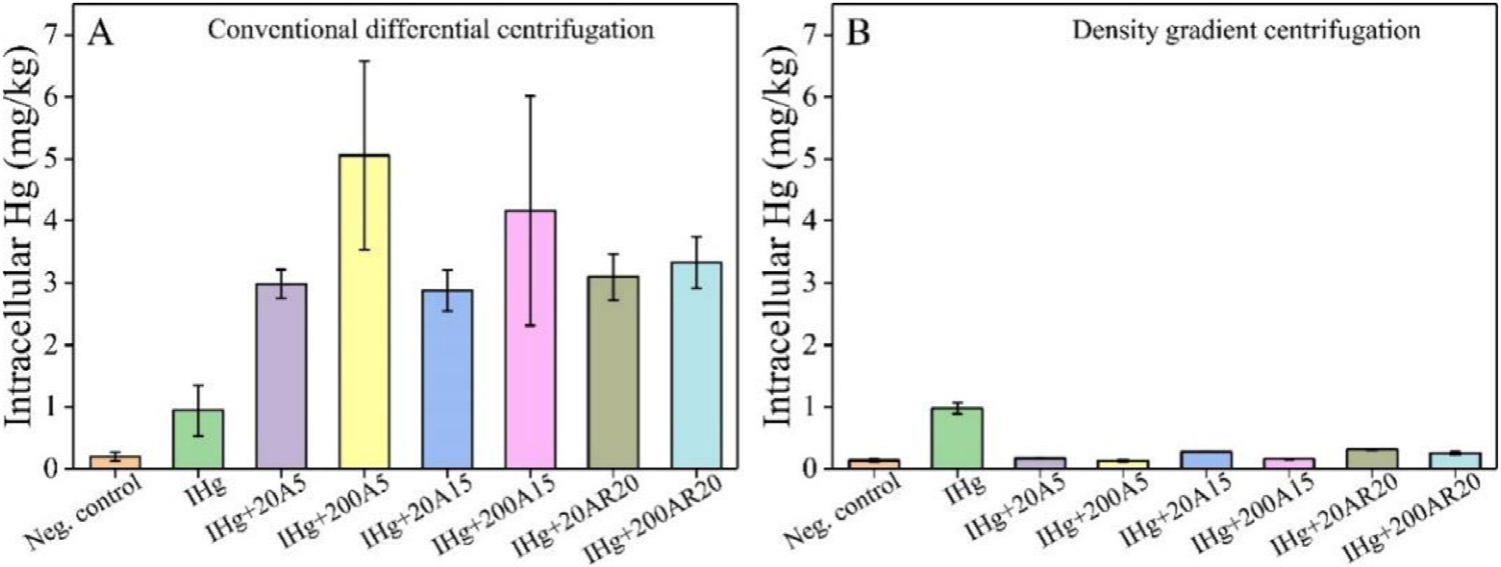
Amount of intracellular (non-extractable by mixture of EDTA and cysteine) Hg in *C. reinhardtii* after the conventional differential centrifugation step (A) and the density gradient centrifugation step (B). Exposure conditions: 10^−9^ M inorganic mercury (IHg) in the absence and presence of 20 or 200 mg L^−1^ of different nanoTiO_2_ materials A5, A15 and AR20, exposure duration 24 h.

In the conventional differential centrifugation method, the presence of nanoTiO_2_ significantly increased the bioaccumulated amount of Hg compared with alga exposed to Hg only. This increase was more pronounced at high nanoTiO_2_ concentrations (Fig. 6A). To be specific, in the treatment of 10^−9^ M IHg, cellular Hg amount in the presence of 20 mg L^−1^ A5/A15/AR20 nanoTiO_2_ was about 3 times higher than in the absence of nanoTiO_2_. In the presence of 200 mg L^−1^ A5/A15/AR20, 5.4, 4.4 and 3.5-time increase of the intracellular Hg was found in comparison with the intracellular Hg amount in the absence of nanoTiO_2._ When density gradient centrifugation method was used (Fig. 6B), the intracellular Hg concentrations in the Hg + nanoTiO_2_ mixture exposure decreased. Intracellular Hg amount in the presence of 20 mg L^−1^ A5/A15/AR20 was around 0.3 times less than the intracellular Hg amount in the absence of nanoTiO_2_. In the presence of 200 mg L^−1^ A5/A15/AR20, intracellular Hg amount was around 0.2 times of the intracellular Hg amount in the absence of nanoTiO_2._ This may be due to the co-sedimentation of the aggregated nanoTiO_2_ with alga. Therefore, neglecting of these effects can lead to significant overestimation of the accumulation of Hg by alga in the presence of TiO_2_ aggregates.

In the present study we used washing of the cells with 10^−3^ M EDTA and 10^−3^ M cysteine (step 5 of the procedure), which was previously shown to allow an extraction of the loosely-bound metals and mercury and allowed to operationally determine the intracellular or non-extractable metal [1]. Therefore, we assume that the possible contribution of Hg bound to the nanoTiO_2_ that could be adsorbed on the algal cells and can not be separated by the proposed methodology will be negligible, since removed by cysteine wash given the strong affinity of Hg to SH- groups. In addition, no significant changes in the SSC and FSC signals of cells were found by FCM, suggesting no measurable adsorption of nanoTiO_2_ to alga after washing with 10^−3^ M EDTA and 10^−3^ M cysteine took place.

## 2 Conclusions

We propose a novel density gradient centrifugation-based method for rapid and efficient separation of green microalga *C. reinhardtii* from nanoTiO_2_ and TiO_2_ aggregates followed by quantitative determination of the Hg accumulated in microalgae co-exposed to nanoTiO_2_ and IHg. A key step of the method is a sucrose gradient centrifugation step allowing to separate efficiently phytoplankton from nanoTiO_2_ and their aggregates and thus to determine the amount of the contaminant that is taken up by the microorganism, avoiding artefacts. The method was developed and validated in a specific case of green microalgae, nanoTiO_2_ and Hg, however, the approach is highly transferable to other ENPs, which do not dissolve significantly, and trace metals in complex environmental mixture.

## Declaration of Competing Interest

The authors declare that they have no conflict of interests.

## References

[1] Beauvais-Flück R., Slaveykova V.I., Cosio C. (2017). Cellular toxicity pathways of inorganic and methyl mercury in the green microalga *Chlamydomonas reinhardtii*. Sci. Rep..

[2] Bowen R., Onge J.S., Colton J., Price C. (1972). Density-gradient centrifugation as an aid to sorting planktonic organisms. I. Gradient materials. Mar. Biol..

[3] Chunxiang L., Jie G., Jianming P., Zhang Z., Yongsheng Y. (2009). Synthesis, characterization, and adsorption performance of Pb (II)-imprinted polymer in nano-TiO_2_ matrix. J. Environ. Sci..

[4] Ghasemi Z., Seif A., Ahmadi T.S., Zargar B., Rashidi F., Rouzbahani G.M. (2012). Thermodynamic and kinetic studies for the adsorption of Hg (II) by nano-TiO_2_ from aqueous solution. Adv. Powder Technol..

[5] Keller A.A., Lazareva A. (2014). Predicted releases of engineered nanomaterials: from global to regional to local. Environ. Sci. Technol. Lett..

[6] Lead J.R., Batley G.E., Alvarez P.J., Croteau M.N., Handy R.D., McLaughlin M.J., Judy J.D., Schirmer K. (2018). Nanomaterials in the environment: behavior, fate, bioavailability, and effects—an updated review. Environ. Toxicol. Chem..

[7] Li M., Liu W., Slaveykova V.I. (2020). Effects of mixtures of engineered nanoparticles and metallic pollutants on aquatic organisms. Environments.

[8] Li M., Liu W., Slaveykova V.I. (2020). NanoTiO_2_ materials mitigate mercury uptake and effects on green alga *Chlamydomonas reinhardtii* in mixture exposure. Aquat. Toxicol..

[9] Mortimer M., Petersen E.J., Buchholz B.A., Holden P.A. (2016). Separation of bacteria, protozoa and carbon nanotubes by density gradient centrifugation. Nanomaterials.

[10] Petrie B., Barden R., Kasprzyk-Hordern B. (2015). A review on emerging contaminants in wastewaters and the environment: current knowledge, understudied areas and recommendations for future monitoring. Water Res..

[11] Sharma V.K. (2009). Aggregation and toxicity of titanium dioxide nanoparticles in aquatic environment—a review. J. Environ. Sci. Health Part A.

[12] Wang Y., He Y., Lai Q., Fan M. (2014). Review of the progress in preparing nano TiO_2_: an important environmental engineering material. J. Environ. Sci..

[13] Zhang L., Liu N., Yang L., Lin Q. (2009). Sorption behavior of nano-TiO_2_ for the removal of selenium ions from aqueous solution. J. Hazard. Mater..

